# Biophysical Mechanisms Mediating Fibrin Fiber Lysis

**DOI:** 10.1155/2017/2748340

**Published:** 2017-05-28

**Authors:** Nathan E. Hudson

**Affiliations:** Department of Physics, East Carolina University, N304 Howell Science Complex, Greenville, NC 27858, USA

## Abstract

The formation and dissolution of blood clots is both a biochemical and a biomechanical process. While much of the chemistry has been worked out for both processes, the influence of biophysical properties is less well understood. This review considers the impact of several structural and mechanical parameters on lytic rates of fibrin fibers. The influences of fiber and network architecture, fiber strain, FXIIIa cross-linking, and particle transport phenomena will be assessed. The importance of the mechanical aspects of fibrinolysis is emphasized, and future research avenues are discussed.

## 1. Introduction

Coagulation and fibrinolysis serve as complementary but competing mechanisms during the process of wound healing. Activation of the coagulation cascade due to vascular injury results in the formation of a fibrin network, which serves to seal the injury. Formation of fibrin activates the fibrinolytic system, a set of enzymes, and inhibitors whose function is to regulate the breakdown of the fibrin network. These systems have been studied for over sixty years, and many of the main pathways have been studied and identified.

In the past decade the mechanical properties of fibrin have received renewed interest with the revelation that fibrin is among the most elastic and extensible biomaterials [1, 2], and recent studies have begun to explore the direct correlation between fibrin extension and fibrinolytic rates [3]. This review will focus on the intersection of fibrinolysis and fibrin's biophysical properties, with an emphasis on basic scientific discoveries and not clinical treatment strategies. However, it is expected that a deeper understanding of how the mechanical properties of fibrin mediate fibrinolysis could have clinical relevance. Lytic strategies for treating acute myocardial infarctions often see recanalization rates of only 80%–90%, while the mechanical breakdown of blood clots often achieves higher patency [4]. This suggests the need for a further examination of the fibrinolytic determinants and highlights the importance of understanding fibrinolysis in light of fibrin's biophysical characteristics. This review is not exhaustive for all aspects of fibrinolysis but emphasizes major events, and as with any review there are many papers that could have been cited that were not and many topics that could have been covered in greater detail that only receive a surface treatment; the author apologizes for any oversites in these cases.

## 2. Fibrinogen and Fibrin

### 2.1. Structure and Polymerization

**Figure 1:**
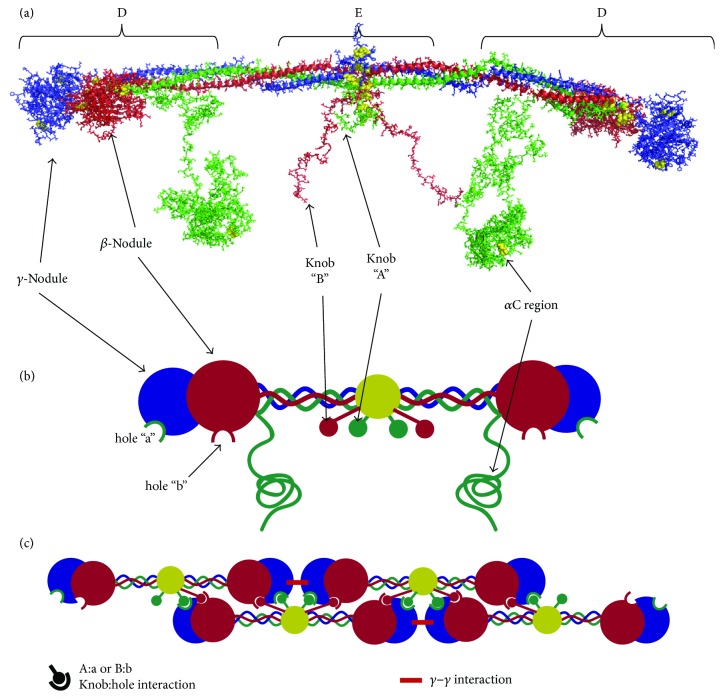
The fibrin molecule and polymerization into fibers. (a) Crystallography-based fibrin molecule: the fibrin molecule structure shown was created using crystal structure 3GHG [9], combined with discrete molecular dynamics methods to fill in amino acids *α*17–26, *α*201–610, and *β*15–57 [15], which were missing in the crystal structure. The *α* chain is shown in green, *β* chain in red, and *γ* chain in blue; disulfide bonds are emphasized as yellow spheres. The *α*C region was built from homology modeling and molecular dynamics methods as described in [15]. Fibrin degradation fragments D and E are highlighted. Fragment X is formed from plasmin cleavage of the *α*C region. (b) Cartoon fibrin molecule: upon thrombin cleavage of FpA and FpB, knob A and knob B are exposed to bind the respective hole a and hole b. Cartoon model highlights these interactions and draws structural correlations between the crystal structure and the cartoon (c) Polymerization model for a protofibril: during polymerization, a half-staggered protofibril is formed as the knobs in the central region of one molecule bind to the holes in the distal region of two opposite molecules. Knob B has been implicated in the lateral aggregation of protofibrils and could potentially bind to holes in adjacent protofibrils (not shown).

Human fibrinogen is a soluble, 46 nm long, 340 kDa glycoprotein and is the third most prevalent protein found in blood plasma, circulating at 6–12 *μ*M [5]. It is assembled as a homodimer, with each subunit consisting of three polypeptide chains (called A*α*, B*β*, and *γ*), having 610, 461, and 411 amino acid residues, respectively [6]. Within the fibrinogen molecule, all six chains are oriented so that their N-termini are located in the central region and held together by five disulfide bonds [6, 7]. From both sides of the central nodule, the three chains extend into *α*-helices that form a triple coiled coil structure, terminating with a series of disulfide bonds, linking the three chains again at the C-terminus of the coiled coil [7, 8]. Beyond this disulfide linkage, the C-terminal segments of the B*β* and *γ* chains fold independently to form the compact, globular *β*- and *γ*-nodules (see Figures 1(a) and 1(b)) [8]. The C-terminal segment of the A*α* chain (called the *α*C region) is different, briefly folding back to form a fourth *α*-helix, before extending into a primarily unstructured region, which has been uncrystallizable [9]. The *α*C region is often grouped into two subregions: the *α*C connector (221–391) and the *α*C domain (392–610) [10, 11]. The *α*C connector region is thought to be unstructured and consists of 10, 13-amino acid repeats [10] in humans, while electron microscopy and circular dichroism (CD) studies indicate that the *α*C domain contains a folded structure [12, 13]. CD and NMR studies have further clarified the *α*C domain structure, finding that the lone disulfide bond in the *α*C domain stabilizes a double *β* hairpin structure in residues *α*392–503, while a second, uncharacterized, structured region also exists in *α*504–610 [13, 14]. A similar, but not identical, *β*-sheet structure was found for the *α*C domain using homology modeling and molecular dynamics simulations [15]. In fibrinogen, it is thought that the *α*C domains interact with each other and the FpB in the central region [16].

**Figure 2:**
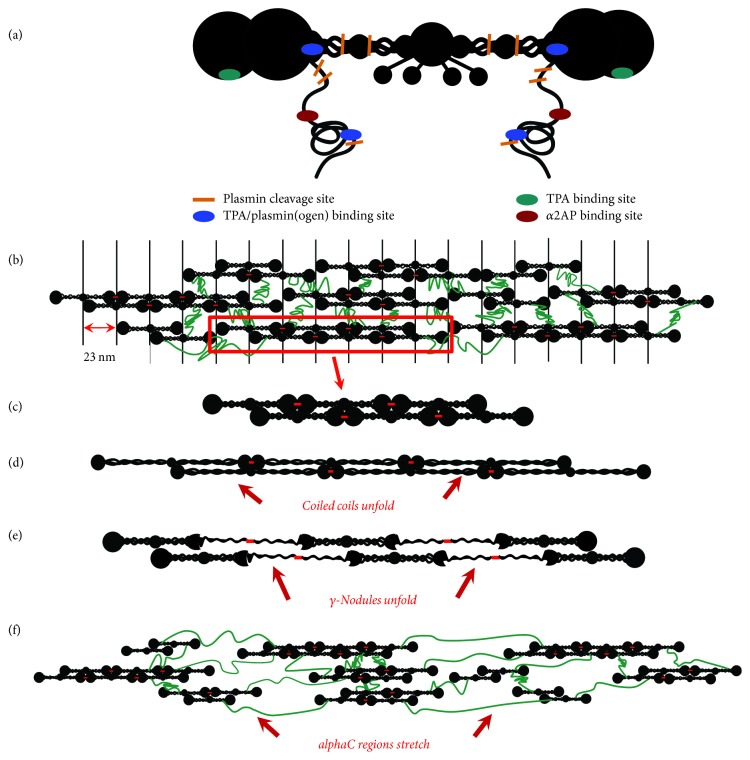
Fibrin fiber structure and mechanical stretching. (a) Cartoon model of the fibrin molecule showing fibrinolytic binding and cleavage sites. Relative positions of plasmin cleavage sites, and tPA, plasmin(ogen), and *α*2AP binding sites are color coded. Mechanical stretching alters each site, as seen below. (b) A structural model for the fibrin fiber, consisting of protofibrils (c) linked together by unstructured *α*C regions. Knob-hole interactions are not shown and the *β*- and *γ*-nodules have been simplified to one structure in (b–f) for clarity. The protofibrils align to give a 23 nm banding pattern as seen in electron microscopy images, although the interactions mediating this alignment are unclear. The red dashes between adjacent molecules indicate the site of *γ*-*γ* FXIII cross-linking. (d–f) Cartoon models depicting extension of the fiber arising from stretching of the coiled coil region (d), *γ*-nodule (e), and the *α*C regions between protofibrils (f), respectively.

Fibrinogen is converted into insoluble fibrin when the enzyme thrombin cleaves the R16-G17 bond in each A*α* chain and the R14-G15 bond in each B*β* chain. Release of these peptides (fibrinopeptides A and B, or FpA and FpB) exposes the “A” and “B” knobs, which bind to corresponding “a” and “b” holes in the *β*- and *γ*-nodules, allowing fibrin fiber polymerization (see Figures 1(a) and 1(b)). FpA is cleaved more rapidly than FpB, and the “A:a” knob:hole interaction is the primary mediator of polymerization [11]. Cleavage of FpB may release the *α*C domains from the fibrin molecule, allowing them to interact intermolecularly [16], and also seems to induce a conformational change in the fibrin molecule [5, 17]. Polymerization proceeds using a half-staggered molecular arrangement in which the knobs in the central region of one fibrin molecule bind to holes in two abutting, nearby molecules [18]. The central region of each of those two molecules also contains knobs, which can bind to two other molecules, and so on. Polymerization propagates in this manner, forming a double-stranded protofibril in the process (see Figure 1(c)). Finally, to form fibers, protofibrils bundle together laterally through interactions between the *α*C regions in adjacent protofibrils (see Figure 2) [5]. Fibers with truncated *α*C regions (A*α*251) display thinner fibers, lower stiffness, and enhanced fibrinolysis, emphasizing the importance of this region of the fibrin molecule [19]. *α*C domain interactions are thought to be mediated by intermolecular *β*-sheet swapping of the *β*-hairpin region [14] and produce high molecular weight digestion products commonly called *α*-polymers that suggest many *α*C regions link together in this manner. *α*-Polymers and the *γ*-nodules in protofibrils are further reinforced by Factor XIIIa (FXIIIa) cross-linking, as discussed later in this report. The resulting product is fibers ranging from eighty to several hundred nanometers thick and 100's of nanometers to 10's of micrometers long [20, 21].

The nanostructure of fibrin fibers has been of longstanding interest. Multiple experimental methods have determined that fibers themselves also contain ~80% water [22, 23], leading to estimates of pore sizes within fibers ranging from 1 to 30 nm [24, 25] and suggesting that fibrinolytic molecules can diffuse even within a fiber [25–27]. Early EM studies on fibrin showed a distinct 23 nm banding pattern across the diameter of fibers, exactly half the length of the fibrin molecule [28]. Later studies showed that protofibrils twist around the exterior of fibers [20]. The banding patterns indicated a lateral registry between protofibrils in the fiber, although the interactions that cause the lateral registry were and still are unclear. A model based on the crystal structures of fibrin(ogen) suggested a quasicrystalline packing of protofibrils inside the fiber with unit cell of dimensions 19 nm x 19 nm × 45 nm [29]. AFM and Small Angle X-ray Scattering (SAXS) data suggested a modified model with a fractal fiber structure still retaining quasicrystalline banding, but having larger distances between protofibrils [23, 30]. Still other studies suggest that the density of protofibrils is not uniform across a fiber diameter and that the protofibril spacing increases as a function of radial distance [20, 31]. Finally, other studies emphasize the flexibility of protofibrils within fibers, which is hard to reconcile with a quasicrystalline packing [32]. None of the models currently include *α*-polymer networks into the packing architecture. More work in this area could shed light on fibrin mechanical properties and the mechanisms of molecular diffusion during fibrinolysis.

Finally, it should be mentioned that while fibrinogen is soluble, polymerized fibrin is insoluble. Thus, although commonly used, Michaelis-Menten kinetics are not quite correct in describing the action of an enzyme on an insoluble substrate. Recent work on fractal kinetics has begun to explore this issue [33].

### 2.2. Plasmin and tPA Binding Sites on Fibrin

Fibrinolytic enzymes including plasminogen and tissue plasminogen activator (tPA) bind to fibrin, and their binding sites will be discussed here, although a detailed description of the fibrinolytic molecules occurs in later sections of this review. Of importance is the fact that fibrin, but not fibrinogen enhances the activation of plasminogen by tPA [34], even though sequence-wise fibrinogen and fibrin only differ by the presence and absence fibrinopeptides A and B. Thus, it has been hypothesized that the conversion of fibrinogen to fibrin causes a conformational change, exposing binding sites for plasminogen and tPA [18].

Intact fibrin has ~100 lysine residues, but no C-terminal lysines. Binding sites have been identified at the periphery of the fibrin molecule for both tPA and plasminogen. The *α* chain residues 148–160 bind both tPA and plasminogen with equal affinity (*K*_*D*_ ~ 1 *μ*M) [35], and a monoclonal antibody raised against the sequence was able to bind fibrin, but not fibrinogen [36]. Electron microscopy studies of plasminogen bound to fibrin also show that it binds to the peripheral “D” region, in agreement with the antibody epitope mapping (see Figure 2(a)) [37]. The binding is lysine dependent, suggesting Kringle domain involvement [38]. A lysine-independent tPA binding site has been localized to *γ* chain residues 312–324 that is also inaccessible to antibodies in fibrinogen, but accessible in fibrin (see Figure 2(a)) [39]. The spatial localization of these sites is in agreement with the observation that a ternary complex between fibrin, tPA, and plasminogen is required to increase tPA's catalytic efficiency [37, 40].

Dysfibrinogenemias with abnormalities in the fibrin *α*C region indicate further binding sites for plasminogen and tPA [41, 42]. To test this, *α*C regions were recombinantly expressed and binding to plasminogen and tPA was measured. Both enzymes bound with high affinity (16–33 nM) [43] to lysine residues in the *α*C domain (*α*392–610), and binding was noncompetitive, suggesting different binding sites for plasminogen and tPA. Other work indicates that the tPA finger domain can bind to cross-beta structures in the fibrin *α*C domain [44]. Thus, there may be multiple bindings sites in the *α*C domain, but not the *α*C connector regions (*α* 221–391) for plasminogen and tPA. The *α*C region also contains binding sites for FXIIIa (*α*389–403) [45] and the cross-linking site for *α*2-antiplasmin (*α*2AP, L303) [46].

### 2.3. Degradation Products and C-Terminal Lysine Binding Sites

At least 34 different plasmin cleavage sites have been identified on fibrin(ogen), but different attack points are cleaved at diverse rates. Because of this, plasmin leaves a series of well-defined fibrin degradation products during lysis. The lytic series was worked out in detail several decades ago and has been reviewed elsewhere [47], so it will only be mentioned briefly here.

The first attack point is A*α*K583, which is partially degraded even in circulating fibrinogen, and the cleavage of which leaves a C-terminal lysine. Subsequent attack points are *α*K206 and *α*K230, which remove nearly the entire *α*C region of fibrin. This *α*C-less fibrin structure was historically called fragment “X” (see Figure 1(a)); the detached *α*C region is further degraded after removal. The next attack region is in the coiled coil, where the *α* (K78, K81, R95, R104, and R110), *β* (K122, K133), and *γ* (K53, K58, K62, K85, and K88) chains all contain 2–5 lysine and arginine residues known to be plasmin cleavage sites. Transection of the coiled coil releases “the D region” containing a portion of the coiled coil and the *β*- and *γ*-nodules (see Figure 1(a)). Upon the cleavage of the other coiled coil, “the E region” is produced, which includes the central region of the molecule containing the N-terminal disulfide knot and a portion of the coiled coil. In FXIIIa ligated fibrin, the D region is covalently cross-linked to an adjacent molecule, and “D-D” and “D-E-D” (where a D-D is noncovalently bound to region E via knob-hole interactions) fragments are released.

The creation of C-terminal lysines in partially degraded fibrin serves as a feedback mechanism for more plasminogen and tPA binding. tPA binding to degraded fibrin is 2–4 orders of magnitude tighter than binding to intact fibrin [48]. Also, removal of the *α*C region eliminates the protection of *α*2-antiplasmin (*α*2AP) from the immediate vicinity of the fibrin molecule, potentially increasing fibrinolysis further.

## 3. Fibrinolytic Agents: Activation and Inhibition

### 3.1. Plasminogen and Plasmin Structure, Conformation, and Function

The primary fibrinolytic agent is the serine protease plasmin. It is a trypsin-like enzyme with broad specificity that cleaves peptide bonds at the C-terminal side of lysine and arginine residues. Plasmin is the activated form of the zymogen plasminogen, which circulates as a single 791 amino acid chain at concentrations of ~2 *μ*M [49]. Several structural features regulate the binding and activity of plasmin(ogen) [the nomenclature plasmin(ogen) will refer to features common to both plasmin and plasminogen].

Plasmin(ogen) has historically been subcategorized, based upon its glycosylation state, into Type I and Type II. Type I has an N-linked glycan at N289 and an O-linked glycan at T346 and comprises 40% of circulating material [50, 51]. Type II only has the O-linked T346 glycan and comprises 60% of circulating material. Subsequent studies suggested that some Type II plasminogen could contain a second O-linked glycan at S248 [52] or S339 [53]. Glycosylation plays a dramatic role in regulating the binding of plasmin(ogen) to particular cell types [54] and seems to play a minor role in the activation of plasminogen on fibrin [55].

The crystal structure of plasminogen was recently determined shedding further light on the structural determinants of plasmin(ogen) function [56, 57]. It can be structurally segregated into an N-terminal Pan-apple domain (PAp; ~1–77), 5-Kringle domains (Kr-1, Kr-2, Kr-3, Kr-4, Kr-5; ~78–542), and a serine protease domain (562–791) [56, 58]. Kringle domains 1, 2, 4, and 5 contain a DXD/E motif for binding C-terminal lysine residues, and lysine binding seems to be the primary mechanism for plasmin(ogen) binding to fibrin and/or cell-surface ligands [58, 59]. In Kringle 3, the motif has been mutated to DXK, and lysine binding is abolished [60]. In the closed conformation (discussed below), only Kr-1 is exposed for binding, suggesting that this domain mediates the initial recruitment of plasmin(ogen) to its binding partners [56, 57].

The PAp domain (sometimes called the N-terminal peptide [57] or activation peptide [58]) plays an important role in regulating plasmin activity and activation. With the PAp attached, plasmin(ogen) (referred to as Glu-plasmin(ogen), in this case) is predominantly found in a compact conformation mediated by an interaction between the PAp and Kr-4/Kr-5 [56, 57]. The compact conformation (typically called the closed conformation) has rough dimensions of 9 nm × 6 nm [37, 57] and a radius of gyration of 3.1 [61] to 3.9 nm [62]. However, plasmin(ogen) can also adopt a much larger U-shaped conformation (the open conformation) with rough dimensions of 14 nm × 7 nm [37] and a radius of gyration of 5.0 [61] to 5.6 nm [62]. Natively there is some thermal equilibrium between the two conformations [63], but the open conformation can be stabilized either through the cleavage of the PAp by plasmin at residue Lys77 or by the binding of lysine or lysine-analogs to the Kr-4/Kr-5 domains. Upon cleavage of the PAp domain, plasmin(ogen) is referred to as Lys-plasmin(ogen). Interestingly, Lys77 is buried in the closed conformation and inaccessible to plasmin, so some conformational rearrangement must occur prior to PAp cleavage [56].

Plasminogen activators cleave the bond between R560 and V561 in the C-terminal region of plasminogen [64], exposing the catalytic triad H603, Asp646, and Ser741 in the serine protease domain. This is the crucial step in the conversion of plasminogen to plasmin and results in a double-stranded plasmin molecule whose two chains are held together by disulfide bonds [65]. The plasmin light chain (~25 kDa) contains the catalytic site, while the heavy chain (~60 kDa) contains the Kringle domains. The R560-V561 bond is shielded from proteolysis by both the Kr-3/Kr-4 loop and the T346 O-linked glycan in the closed conformation of plasminogen [56]. However, the open conformation has a 3–50 fold increased rate of activation [63], suggesting that R560-V561 shielding is greatly reduced in this conformation.

Thus, there is a direct connection between the conformation of the plasminogen molecule and its ability to be activated to plasmin. Because the plasminogen conformation is governed by the interaction between PAp and Kr-4/Kr-5, cleaving the PAp domain or plasminogen binding to C-terminal lysines, as is the case when binding to fibrin or cell receptors, will greatly enhance the conversion to plasmin. Additionally, because Glu-plasminogen can bind to ligands prior to PAp cleavage, and binding promotes activation, it is possible to have Glu-plasmin, in addition to Glu-plasminogen. However, higher local plasmin concentrations lead to higher catalytic rates of PAp cleavage, so the predominant activation pathway is Glu-plasminogen → Lys-plasminogen → Lys-plasmin.

### 3.2. Plasminogen Activators (PA's)

The two primary physiological plasminogen activators are the serine proteases urokinase-type plasminogen activator (uPA) and tissue-type plasminogen activator (tPA) (for more detailed reviews see [65–68]). While having similar catalytic function, differences in the binding domains of uPA and tPA result in differentiation in localization and different biological roles for the two PA's [66]. Other potential physiological plasminogen activation pathways, such as the contact activation pathway will not be covered in this review [68, 69].

#### 3.2.1. uPA

uPA is secreted as a single-chain, 411-amino acid, protein (sc-uPA) that has very little intrinsic catalytic activity in plasma [70, 71]. uPA activation transpires from the proteolysis of L158-I159 bond, converting sc-uPA to two-chain uPA (tc-uPA) and exposing the serine protease site [72]. tc-uPA also has a low molecular weight form, created via plasmin cleavage of the L135-L136 bond [69, 73], which circulates in plasma, but most circulating sc-uPA and tc-uPA are cleared from plasma within minutes [74].

uPA consists of three structural regions, a C-terminal serine protease domain (159–411), a Kringle domain (50–132), and a growth factor domain (GFD; 10–43) [75]. Unlike tPA, uPA's Kringle domain does not have a fibrin binding site, and uPA has low affinity for fibrin [69]. uPA binds tightly (*K*_*D*_ < 1 nm) [76] to cell-surface receptor urokinase-type plasminogen activator receptor (uPAR) through its GFD [77, 78], although it also binds and activates plasminogen on platelets, which do not express uPAR [79]. Interestingly, sc-uPA shows ~100 fold increase in activity when bound to cell surfaces, while tc-uPA's activity is not increased further by cell binding [79, 80].

uPA primarily activates cell-surface bound plasminogen, although it can also activate solution-phase plasminogen, in contrast to tPA [68]. Surface-activated plasmin plays an important role in extracellular matrix degradation and growth factor activation [81]. The precise role uPA plays in fibrinolysis is still controversial; however, mouse models show an active role for uPA in fibrinolysis [82, 83] and tc-uPA activates Glu-plasminogen at a 10-fold higher rate in the presence of fibrin in spite of not binding to fibrin [69], so uPA's role in fibrinolysis should not be minimized.

#### 3.2.2. tPA

tPA is synthesized and secreted by endothelial cells as a single-chain, 527-amino acid, glycoprotein. The plasma concentration of tPA is 70 pM, and it has a half-life of 4 minutes, so it is tightly regulated [68, 84]. Unlike other serine proteases like uPA and plasmin, the single chain of tPA (sc-tPA) has inherent catalytic activity and can activate plasminogen [85]; however, cleavage of the R275-I276 bond by plasmin and conversion to two-chain tPA (tc-tPA) increases plasminogen activation rates from 3- to 10-fold in the absence of fibrin [34, 40]. In the presence of fibrin, the activity of tPA is increased from 100- to 1000-fold and sc-tPA and tc-tPA have comparable catalytic rates [34, 40, 86]; the presence of fibrinogen does not increase tPA activity [18, 40]. There is strong evidence that the rate enhancement occurs due to the formation of a ternary complex between fibrin, plasminogen, and tPA [37, 40].

Fibrin stimulation of plasminogen activation by tPA occurs in two phases [87–89]. The first phase is mediated by the conversion of fibrinogen to fibrin and the exposure of cryptic tPA and plasminogen binding sites on fibrin. During this phase the typical tPA* K*_*m*_ value is ~1 *μ*M plasminogen and the catalytic rate constant is ~0.2 s^−1^ [40, 89]. Upon plasmin formation, COOH-terminal lysines become exposed as fibrin is digested; this provides more binding sites for plasminogen and tPA and creates a positive feedback mechanism, resulting in* K*_*m*_ values for tPA ~ 100 nM plasminogen while retaining the same catalytic rate constant [89]. For these reasons, tPA is thought to be the predominant plasminogen activator during fibrinolysis.

Structurally tPA consists of 5 distinct domains: a finger domain, an epidermal growth-factor-like domain, two Kringle domains, and the catalytic domain [90]. The finger domain and the Kringle-2 domain serve as the primary fibrin binding sites [86]. The Kringle-2 domain plays a role in C-terminal lysine binding, while the finger domain can bind to a region in the fibrin *γ*-nodule in a lysine-independent mechanism [91] or to amyloid-like cross-beta structures [44], which have been hypothesized to form in fibrin *α*-polymers [14, 92]. Recent work has shown that the tPA finger domain plays the predominant role in binding to fibrin during fibrinolysis [93, 94], even in proteolytically degraded fibrin.

Fibrin thus acts as both a cofactor and substrate for tPA and plasminogen and in so doing provides the mechanism for its own disintegration. These dual roles highlight the intended temporariness of the fibrin mesh network; it is not designed to stick around for longer than is necessary for wound healing in most physiological circumstances.

### 3.3. Fibrinolytic Inhibitors

The proteases of the fibrinolytic system are all tightly controlled by inhibitors. For recent, thorough reviews, the reader is directed to [68, 95]. For the purposes of this review, they will only be covered briefly.

#### 3.3.1. *α*2-Antiplasmin (*α*2AP)

*α*2AP serves as the fast-acting (4 × 10^7^ M^−1^s^−1^) [96] primary inhibitor of plasmin and circulates in plasma at concentrations of ~1 *μ*M, usually in excess of plasmin, with a half-life of 3 days [68, 97]. *α*2AP is also cross-linked to fibrin by FXIIIa, so there is a high local concentration in clots (this is described in more detail later in this review) [98], and there are additional noncovalent *α*2AP binding sites in fibrin, but not fibrinogen [99]. Like other serpin inhibitors, *α*2AP inhibits plasmin by inserting a “reactive center loop (RCL)” into plasmin's catalytic site, which then attacks the R364-M365 peptide bond of the loop [100]. This releases the N-terminal portion of *α*2AP, while forming a covalent ester-bond between *α*2AP and the catalytic site, inhibiting plasmin. The lysine-rich C-terminus of *α*2AP is ~55 residues longer than most serpins and contains a binding site for plasmin(ogen) Kringle domains [97, 101]. Plasminogen-fibrin and plasminogen-*α*2AP binding are competitive; plasmin activated while bound to fibrin is therefore relatively protected from *α*2AP, although the unbound *α*2AP can still inhibit plasmin albeit at a 100-fold slower rate [100].

#### 3.3.2. Plasminogen Activator Inhibitors 1, 2 (PAI-1 and PAI-2)

PAI-1 is the physiological inhibitor of both uPA and tPA. Like *α*2AP, PAI-1 is a serpin inhibitor, with its reactive site at R346-M347 [95]. PAI-1 inhibits both tPA and tc-uPA with second-order rate constants of roughly 1−4 × 10^7^ M^−1^s^−1^ [102, 103]. Activated platelets can release PAI-1 and increase its local concentration 10-fold [104], helping to reduce fibrinolysis at the onset of clotting.

PAI-2 also is a serine protease that inhibits tPA (1 × 10^4^ M^−1^s^−1^) and uPA (2 × 10^6^ M^−1^s^−1^), but with slower rate constants than PAI-2 [105]. Its primary function may be related to placental maintenance rather than fibrinolysis, as it is only present in plasma during pregnancy [68, 95].

#### 3.3.3. Thrombin-Activatable Fibrinolysis Inhibitor (TAFIa)

TAFIa is not a serine protease and has an entirely different mechanism than other fibrinolytic inhibitors [106]. TAFIa is produced from its zymogen TAFI by cleavage at R92-A93 by the thrombin/thrombomodulin complex [107]. TAFIa removes C-terminal lysine and arginine residues from fibrin as it is degraded, preventing plasminogen binding and the positive feedback mechanisms that stimulate lysis [108]. It also prevents the conversion of Glu- to Lys-plasminogen, reducing the rate of plasminogen activation [109]. Thus, TAFIa does not directly inhibit lysis, but rather slows down several crucial steps in fibrinolytic upregulation.

## 4. Biophysical Determinants of Fibrinolysis

### 4.1. Clot Structure and Architecture

Blood clot content and architecture help to determine their lytic susceptibility. Fibrin network structure is determined by the local concentrations of fibrinogen, thrombin, and ions, such as Ca^2+^ [110, 111]. High fibrinogen concentrations, such as those experienced in hyperfibrinogenemia, high local thrombin concentrations, and high plasma ionic strength give rise to clots with thinner fibers at a higher density (less space between fibers) [110–113]. Conversely, increasing the Ca^2+^ concentration or decreasing the thrombin concentration leads to clots composed of thicker fibers and a lower packing density [110]. A long history of rheological studies suggest that clots composed of thin, densely packed fibers are stiffer than those of thick fibers with larger pore sizes [110, 114, 115].

The mechanism by which individual fibers are lysed has been a subject of debate. Several studies, primarily using turbidity as a readout of fibrinolysis, and models suggested that the diameter of fibrin fibers decreases uniformly during lysis due to many plasmin molecules binding and digesting the fiber along its entire length [116–118]. It should be noted that using turbidity to measure lytic rates has been a subject of controversy with respect to whether the data should be normalized by the highest turbidity value [119]; doing the normalization lowers the apparent lytic rates after polymerization reaches its maximum but ignores digestion prior to that point. Other studies, primarily using fluorescent and electron microscopy reported transverse cleavage of fibers at one point [120–123]. Because C-terminal lysines are exposed during lysis, plasminogen binding to partially degraded fibrin will be amplified at points where fibrin has already been cleaved [124]. It has been hypothesized that this serves as a feedback mechanism to promote further lysis at those points, leading to transverse cleavage directly across the diameter of the fiber [26]. Studies of individual fiber lysis showed that cleavage does occur at a specific point but also that the lytic rate for further fiber degradation slows after the initial transverse cleavage event [121]. It is often observed that cleaved fibers bundle together to form thicker fibers [120, 123] prior to being degraded further. It is likely that both transverse cleavage and digestion along the fiber length play a role during fibrinolysis, perhaps with transverse cleavage mediating the initial digestive event.

The conventional wisdom has been that fiber density (the number of fibers per unit volume) and fiber thickness have competing effects on fibrinolysis. Numerous in vitro studies have reported that clots composed of thinner, more closely spaced fibers, are more resistant to fibrinolysis [111, 112, 120, 125]; however, several studies have shown an opposite effect, so this is not necessarily always the case [116, 126]. Hypofibrinolysis has been reported for patients with thin, dense fibers, supporting the idea that clots composed of thinner fibers are more resistant to lysis [127, 128]. Conversely, thin fibers were more rapidly cleaved than thick fibers in a variety of fibrinolysis models [120, 121, 123, 126]. These contrasting results likely come from the interplay between the movement of fibrinolytic agents (plasminogen, plasmin, tPA, etc.) within a clot, and the activity of those agents on fibers upon binding. It is important to note that, in most in vitro studies of fibrinolysis, plasmin or plasminogen activators such as tPA or uPA are added from outside an already formed fibrin network. Therefore, the permeation of fibrinolytic agents into a clot plays a predominant role in determining clot lysis rates. These studies are important for the development of therapeutics that must be administered from outside the clot but may not mimic in vivo fibrinolysis which happens concurrently with polymerization [123].

In the case where lytic agents are released from outside the clot, recent 3D stochastic modeling suggests that the determining factor in fibrinolytic rates is the number of tPA molecules per clot surface area [27]. Bannish et al. found that, for low tPA concentrations, clots of thick fibers lyse more rapidly, but for higher concentrations (high enough for at least one tPA molecule to bind to every fiber on the surface of the clot), clots composed of thinner fibers will actually lyse faster than those of thick fibers [27]. These results may help to explain the discrepancies between previous experiments and suggest avenues for therapeutic development.

The observation that thicker fibers lyse more slowly has been explained, in part, by several phenomena. First, thin fibers are composed of fewer protofibrils within a cross-section, so fewer molecules need to be cleaved in order to transect a fiber [26, 27]. Also, within thin fibers the molecules are more densely packed, so plasmin and tPA binding sites are closer together [31], and it has been observed that thin fibers are better for plasminogen activation by tPA [93]. Therefore, all other things being equal, thin fibers should lyse more rapidly than thick fibers. Secondly, thicker fibers are likely under more tension than thinner fibers due to protofibril packing [20]. Modeling and experiments show that, as fibers are lysed, they lose this inherent tension leading to elongation, and elongation hinders fiber lysis [121]. Elongation is more prominent in thicker fibers than thin fibers providing additional reasons that thin fibers lyse more rapidly. Finally, models predict that the amount of time tPA remains bound to fibers can have a noticeable influence on lytic rates of individual fibers, and tPA remains bound longer to thicker fibers than thin ones independent of the tPA off-rate, if the off-rate is sufficiently slow [27].

### 4.2. FXIIIa Cross-Linking

The transglutaminase FXIIIa likely regulates fibrinolysis through at least three distinct mechanisms: (1) cross-linking fibrinolytic inhibitors, particularly *α*2-antiplasmin, to fibrin, (2) cross-linking fibrin fibers, and (3) altering the mechanical properties of fibers and fibrin networks.

FXIII is a protransglutaminase consisting of two A and B subunits (A_2_B_2_) in plasma and as a homodimer of A subunits (A_2_) in cells [129]. Plasma FXIII is activated in the final step of the clotting cascade when thrombin hydrolyzes the R37-G38 bond, releasing an activation peptide, and Ca^2+^ causes the dissociation of the B subunit, resulting in a catalytically active A_2_ dimer usually referred to as FXIIIa. The rate of plasma FXIIIa activation is accelerated 6-fold in the presence of polymerized fibrin [130]. Cellular FXIII, such as that released by platelets and monocytes, is activated in a thrombin- and fibrin-independent mechanism involving Ca^2+^, where the activation peptide is not removed [131].

It now seems clear that cross-linking *α*2-antiplasmin (*α*2AP) to fibrin is the primary antifibrinolytic function of FXIIIa [98, 132]. *α*2AP plays several inhibitory roles in fibrinolysis including rapidly inhibiting plasmin and interfering with the binding of plasminogen to fibrin lysine sites [46, 133]. During fibrin polymerization, FXIIIa covalently cross-links *α*2AP via its Q2 residue to L303 in the fibrin *α*C linker region [46, 134]. *α*2AP cross-linking precedes *α* chain cross-linking by FXIIIa (see next paragraph) and may inhibit this process [129, 133]. Uncross-linked *α*2AP has similar plasmin inhibitor activity to cross-linked *α*2AP, but cross-linked *α*2AP has a much greater effect on the inhibition of lysis [135]. This inhibitory effect is increased during platelet retraction, when the fibers of the clot are closer together and fluid is expelled from the clot [132, 135]. FXIIIa can also cross-link other fibrinolytic inhibitors to fibrin(ogen), including PAI-2 [136] and TAFI [137]. These results strongly support the hypothesis that FXIIIa inhibits fibrinolysis by the covalent incorporation of fibrinolytic inhibitors into the fibrin network. This may be particularly important during the early stages of clot formation, protecting against immediate elimination of nascent clots [129]. However, other studies have shown that *α*2-antiplasmin works in concert with *α* chain cross-linking in fibrinolytic inhibition [138], so FXIIIa cross-linking of fibrin itself likely also has inhibitory effects.

During fibrin polymerization, FXIIIa forms *γ*-glutamyl- *ε*-lysyl cross-links between residues in the *γ* and *α* chains of fibrin monomers. FXIIIa first catalyzes the formation of isopeptide bonds between *γ*1L406 and *γ*2Q398 or *γ*2Q399 at the C-terminal *γ*-nodules of adjacent molecules, forming longitudinal *γ*–*γ* dimers within protofibrils [139]. Later during polymerization, FXIIIa targets lysine and glutamine residues in the *α*C region, resulting in the formation of high molecular weight fibrin species including *α*-polymers and *α*-*γ* hybrids [140–142]. Although there is no set order in which *α* chain glutamine residues are cross-linked, generally Q237 is targeted first, followed by Q366, Q328, and Q221 [143]. The *α* chain lysine donors are more heterogeneous but involve at least L418, L508, L539, L556, L580, and L601, most of which are located at the C-terminal periphery of the *α*C region [144–146]. FXIIIa cross-linking causes slight, but not dramatic changes in network morphology, with 10% thinner fibers, and a 2-fold reduction in pore size [110, 147].

Whether FXIIIa cross-linking effects fibrinolytic rates was a subject of historical [148–150] and even recent [98, 147] debate, complicated by sample preparation and the presence of fibrinolytic inhibitors. A recent study, where *α*2-antiplasmin was inhibited, established that FXIIIa cross-linked fibers have delayed fibrinolysis, even in the absence of external mechanical force [147]. These results agreed with previous studies showing decreased lysis by plasmin on FXIIIa ligated clots [151]. This may be due, in part, to the decreased binding affinity of plasmin or plasminogen to cross-linked fibers [126, 129]. Some studies show that specifically *α* chain cross-linking plays an important role in reducing the fibrinolytic susceptibility of clots [140, 149], although there is not universal agreement [147, 148]; it seems feasible because *α* chain cross-linking likely reduces the number of lysines available for plasminogen and tPA binding, decreases the mobility of molecules between protofibrils [129], and makes protofibril packing more dense [152]. Other studies have shown a predominant role for *γ*-cross-linking and even *γ*-multimers in regulating fibrinolytic rates [153]. In summary, it seems that cross-linking of fibers plays a measurable, but potentially minor role in fibrinolysis.

FXIIIa cross-linking has a dramatic effect on fibrin mechanical properties. Uncross-linked or partially cross-linked (some low molecular weight species) fibrin fibers are among the most extensible biomaterials found in nature, able to be stretched to triple or quadruple their original length before failing and also able to relax back to their original length within milliseconds [1, 15, 154, 155]. Fully cross-linked fibers (>90% *γ* and *α* chains cross-linked) are 2–10 times stiffer, 50% less elastic, and have 40–50% lower extensibility than partially cross-linked fibers [21, 155]. Studies using recombinant fibrin with *γ* chain cross-linking sites mutated out (*γ*Q398N/Q399N/K406R) reveal that loss of fiber elasticity and extensibility is primarily due to *α* chain cross-linking [156]. Loss of fiber extensibility may explain the recent observation that during clot retraction erythrocytes are trapped in FXIIIa cross-linked fibrin networks without being covalently bound to the fibers [157, 158], while erythrocytes in uncross-linked fibers were extruded.

Fibrin networks also have remarkable extensibility, due in large part to the mechanical properties of their individual fibers [2, 32]. In rheological studies, where network mechanical properties typically depend more on fiber structural rigidity and network rearrangement than fiber stretching, networks composed of cross-linked fibers also exhibited a 2- to 5-fold higher elastic modulus (stiffness) and a 2-fold higher loss modulus [21, 141, 152, 159]. Rheological measurements on (*γ*Q398N/Q399N/K406R) fibrin suggest that FXIIIa-mediated stiffening comes from contributions of *α*- and *γ*-cross-linking, with *α* chain cross-linking playing the largest role [141]; other studies demonstrate that the mechanism of network stiffening comes from FXIIIa-mediated structural rigidity increases of individual fibers [152].

The effect of different mechanical properties in FXIIIa-cross-linked fibrin on fibrinolytic rates has not been directly explored, but several mechanisms can be proposed. First, the reduced extensibility of FXIIIa cross-linked fibers limits the extension of fibers during platelet retraction. Platelets carry endogenous FXIIIa, so most fibers are highly cross-linked during retraction, and because retraction plays multiple roles in regulating fibrinolysis (discussed below), it is likely that the increased stiffness of FXIIIa fibers has a mechanism in this regulation. Second, Varjú et al. showed that fiber stretching decreased plasminogen activation and lysis, suggesting that stretching of fibrin alone regulates fibrinolysis [3], and thus reduced extensibility of fibrin by FXIIIa should affect fibrinolytic rates via this mechanism as well. Third, under certain conditions, thick fibers elongate during lysis and reach a lysis resistant state [121]. FXIIIa cross-linking could alter the elongation and lytic resistance of these fibers. While only hypotheses at this point, these ideas highlight the need for further studies to measure the direct effect of altered fibrin mechanical properties on the fibrinolytic susceptibilities of fibrin clots.

Recently it was shown that plasmin can inactivate FXIIIa, by cleaving the enzyme at a variety of sites, predominantly the K468-Q469 bond [160]; contrastingly, FXIII (A_2_B_2_) was not degraded in the same manner. FXIIIa inactivation by plasmin occurs primarily during fibrinolysis rather than polymerization suggesting it serves as a feedback mechanism to prevent further FXIIIa activity after the cessation of clotting. This could also avert the further incorporation of fibrinolytic inhibitors such as *α*2AP into the clot, thus promoting fibrinolysis.

### 4.3. Movement of Fibrinolytics into and within Fibrin Networks

The transport of fibrinolytic agents into a clot and their movement within a clot depend on diffusion (the random movement of molecules due to thermal fluctuations), advection (the conveyance of particles within flowing fluid; sometimes referred to as convective transport, permeation, or perfusion), and binding (to fibrin or other clot constituents like platelets or erythrocytes). Fibrinolytic transport has been covered in other reviews [24, 161]; the physical aspects of this process will be reviewed here.

Penetration of fibrinolytics into blood clots depends on the network architecture and contents. Networks formed of purified fibrin have a fibrin content usually <1% of the total network volume at physiological fibrinogen concentrations [161]. Under these conditions, the average pore size (space between fibers) ranges from 100 nm in gels made of thin fibers to 10 *μ*m in gels made of thick fibers [24]. The diffusion of a molecule such as plasmin(ogen), with a stokes radius of ~5 nm, within the pores between fibers and cells can roughly be thought of as free diffusion [24, 161].

Cells, such as erythrocytes and platelets, modulate network structure through direct fibrin-cell receptor binding and the release of pro- and anticoagulation factors [162, 163]. Moreover, tissue-factor bearing cells promote fibrin production and can lead to high local fibrin concentrations during polymerization [125]. High local fibrin concentration (up to 400 *μ*M) could decrease the fiber pore size to as low as 4 nm [164], but this is likely not the case under most physiological situations. Even for platelet retracted clots, where 99% of the fluid volume has been expelled, the porosity is still >90% [24]. Taken together, these data suggest that under most conditions, the diffusion of fibrinolytic molecules into and within a clot should roughly mimic free diffusion [165]; however, a recent report on fibrinolysis of stretched fibrin clots reported hindered diffusion into the clot based on Fluorescence Recovery after Photobleaching (FRAP), so this might not always be the case [166].

Although network structure usually does not hinder diffusion, the binding of fibrinolytic molecules to fibrin or cellular constituents plays a dramatic role in reducing their mobility [24, 27, 161]. Fluorescent microscopy studies on the lysis of clots initiated by adding lytic agents outside the clot often show a “lysis front” where plasmin, plasminogen, or tPA bind to the first few microns of a clot without penetrating much further [120, 124]. The network is dissolved progressively from outside to inside as the lysis progresses. Streptokinase, which does not bind tightly to fibrin, penetrates clots more rapidly than tPA [167], and studies using a tPA variant defective in fibrin binding also observed more rapid penetration into clots [93]. Networks composed of thin, densely packed fibers, have more binding sites per unit volume for plasminogen and tPA, which helps to explain the hindered fibrinolysis for these types of clots [27, 120].

Molecular penetration into clots and fibrinolysis rates can be enhanced from 10- to 100-fold by the presence of flow and molecular advection [118, 168, 169]. Flow allows fibrinolytics to travel further into a clot prior to binding, enhancing the inner-clot fibrinolytic rate. The direction of flow matters as tangential flow with respect to the clot can lead to a “plasmin steal” effect where flow depletes plasminogen from the clot boundary [124]. However, a study on retracted blood clot dissolution under tangential flow still showed a 10-fold increase in clot degradation [170]. In the case of flow directly into a thrombus, the fluid will flow through the least-permeation-resistant path, so structural heterogeneity can have dramatic effects on the delivery of fibrinolytics [118, 169]. As the fibrin network is digested, channels will be carved out, and further digestion will emanate outward from the channel [124, 171]. In the case of a completely occluded blood vessel, once a channel is carved through a thrombus, reperfusion of the channel is achieved. The accompanying drop in pressure can reduce the transport of further fibrinolytics into the clot due to flow, and further lysis must proceed through diffusion and binding, as described above. The difference in transport in arterial and venous flow rates can have a dramatic difference in the impact of advection on fibrinolytic rates [161], and therapeutic strategies should be designed accordingly.

### 4.4. Fiber Stress and Extension

Fibrin is among the most extensible biomaterials [1, 172]. Fibrin's elasticity and extensibility may play prominent roles during blood clot formation under shear stress [3, 173–175] and during platelet retraction [176, 177]. The molecular mechanisms underlying fibrin extensibility have recently been debated (see Figures 2(d)–2(f)) [178]. Fiber extension measurements, simulations, and a comparison of human, mouse, and chicken fibrin extensibilities all suggest that the *α*C connector region plays a large role in fibrin elasticity [32, 154, 156, 179, 180]. Other measurements and simulations suggest that unfolding of either the coiled coil region [2, 181–183] or the *γ*-nodule could play roles [15, 182, 184]. Because plasmin(ogen) has potential binding and cleavage sites in each of these regions, fibrin stretching could act as a modulator for fibrinolysis.

During clot retraction (contraction) fibrin fibers are stretched by platelets. This reduces the interstitial spacing between fibers, reduces clot volume, and expels up to 99% of the liquid from within the clot [185]. Retraction may also help to segregate red blood cells (erythrocytes) and form a more effective wound seal [186]. For many years, it has been observed that lysis is altered by clot retraction, and the consensus of most in vitro studies is that platelet retraction inhibits fibrinolysis [185, 187–191]; however, the effects and mechanisms have been debated. One proposal is that the expulsion of unbound plasminogen during retraction reduces lytic rates [185, 192, 193]. tPA binding to fibrin also is inhibited by retraction and may play a larger role than plasminogen depletion in hindering fibrinolysis [189, 191]. Other studies suggested that the increased concentration of FXIIIa-cross-linked *α*2-antiplasmin in retracted clots resulted in higher plasmin deactivation and slower lysis [132, 194, 195]. Studies on the effects clot retraction on fibrinolysis are complicated by the fact that activated platelets contain and/or release a number of hemostatic and fibrinolytic factors including fibrinogen, FXIIIa, plasminogen, plasminogen activators, *α*2AP, and PAI-1, so it is likely that platelets and retraction have multiple roles in regulating fibrinolysis [190, 194, 196, 197].

Several studies have looked at the direct impact of fibrin strain on lysis rates. A study by Varjú et al. attempted to directly measure the fibrinolysis of stretched networks of fibers in the absence of platelets and found that the digestion of fibers formed under mechanical stress was delayed [3]. Plasminogen activation by tPA decreased by 2- to 3-fold on stretched fibers as compared to unstretched. The digestion of stretched surface fibers by both tPA activated plasminogen, and by the direct addition of plasmin, showed a greater than 50% reduction in lysis at comparable time points when compared to unstretched fibers. Another study by Adhikari et al. found a 10-fold reduction in plasmin degradation of strained clots and correlated this with a reduction of diffusive transport into the network [166]. These results suggest that fiber stretching impairs fibrinolysis by delaying plasminogen activation, reducing the fibrinolytic ability of plasmin, and hindering the entrance of fibrinolytics into the network.

In contrast, a study on the lysis of individual, isolated, unstretched fibrin fibers by plasmin showed that as fibers are lysed, they lose their inherent tension and elongate [121]. Elongated fibers reached a state where further fibrinolysis was impaired and often were not further digested. The effect was dependent on fiber diameter, with thicker fibers more likely to elongate, but independent of plasmin concentration. The results suggested that a minimum fiber tension may promote plasmin activity [121]. Because fibrin fibers form under tension [20, 198], one resolution is that polymerization tension is required for fibrinolysis, but the addition of external tension, such as in the case of retraction, hinders fibrinolysis.

Taken together, these results suggest that fiber tension and stretching play an important role in the regulation of fibrinolysis, altering the binding of plasminogen activators, the availability of fibrinolytic enzymes, and the activity of plasmin. Models of fibrin extension often rely on protein unfolding to correlate extensibility with molecular structure [2, 184]. Unfolding of the coiled coil or *γ*-nodule or stretching of *α*C domains [154] could alter or partially block enzyme binding and cleavage sites. Further studies could help to decouple the roles between these different effects.

## 5. Conclusions

**Figure 3:**
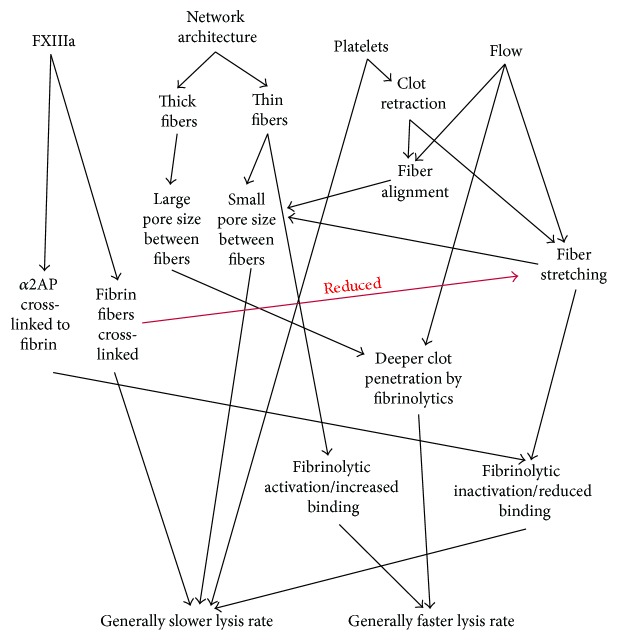
Interaction network of the biophysical determinants of fibrinolysis rates. A diagram highlighting the influence of FXIIIa, network architecture, platelets, and fluid flow on lytic rates. The diagram is simplified and does not include many of the interactions discussed in the paper but is meant to emphasize some of the major impacts. Black arrows show an influence of one property on a downstream property. The end result is either faster or slower network fibrinolytic rates. The red arrow indicates that cross-linked fibrin fibers have reduced extensibility and thus reduced fiber stretching. “Fibrinolytic activation/increased binding” and similarly worded effects are meant to indicate that a fibrinolytic enzyme such as plasmin is activated and/or has increased binding affinity or avidity.

Coagulation and fibrinolysis are very physical processes, performed amid fluid flow, cellular adhesion, and platelet contraction. This review has highlighted several biophysical mechanisms that regulate fibrinolytic rates (see Figure 3). Additional work in this area is needed to understand the mechanisms undergirding the delayed lytic rates of strained fibrin, given that platelet retracted clots contain almost exclusively stretched fibrin fibers. Improved understanding of the connection between the biophysical aspects of fibrin and fibrinolytic rates could lead to new strategies in the development of future fibrinolytic therapies [199].
